# Differences in visuospatial cognition among table tennis players of different skill levels: an event-related potential study

**DOI:** 10.7717/peerj.17295

**Published:** 2024-05-30

**Authors:** Kuan-Fu Chen, Ting-Yu Chueh, Tsung-Min Hung

**Affiliations:** Department of Physical Education and Sport Sciences, National Taiwan Normal University, Taipei, Taiwan

**Keywords:** Cognitive function, Sport, Elite, Event-related potential

## Abstract

This study aimed to examine the influence of sport skill levels on behavioural and neuroelectric performance in visuospatial attention and memory visuospatial tasks were administered to 54 participants, including 18 elite and 18 amateur table tennis players and 18 nonathletes, while event-related potentials were recorded. In all the visuospatial attention and memory conditions, table tennis players displayed faster reaction times than nonathletes, regardless of skill level, although there was no difference in accuracy between groups. In addition, regardless of task conditions, both player groups had a greater P3 amplitude than nonathletes, and elite players exhibited a greater P3 amplitude than amateurs players. The results of this study indicate that table tennis players, irrespective of their skill level, exhibit enhanced visuospatial capabilities. Notably, athletes at the elite level appear to benefit from an augmented allocation of attentional resources when engaging in visuospatial tasks.

## Introduction

Existing research demonstrates that long-term and intensive training in sports, particularly among elite athletes, leads to not only an enhancement in athletic competencies but also an elevation in cognitive function (Logan et al., 2022). These athletes manifest sophisticated multi-dimensional cognitive proficiencies, including aspects of perception, attention, memory, anticipation, and decision-making (Mann et al., 2007; Furley & Memmert, 2011; Ericsson et al., 2018). Yet, numerous studies employing sport-specific tasks have revealed that, although athletes may demonstrate superior sport-related cognitive performance (Jin et al., 2011; Wang & Tu, 2017), the effects on fundamental cognitive faculties remain non-significant (Furley & Memmert, 2011; Ericsson et al., 2018). Importantly, scant research has delved into the extent to which cognitive expertise in sports is transferable to non-sport cognitive tasks, or whether such benefits encompass measures of cognition that align with underlying cognitive demands (Voss et al., 2010). Recent neuroimaging investigations have provided evidence that sustained professional skill training among elite athletes may induce plasticity in both brain structure and function (Zhang et al., 2021), shedding light on the neural mechanisms engendered by long-term sports training. Thus, the present study endeavors to explore the cognitive advantages accrued from prolonged training in elite athletes (high-skill performance), focusing on sport-specific domains, while examining the potential transference of these benefits to non-sport cognitive domains, and delineating the neural processes activated in response to information processing demands.

Athletes’ visuospatial ability and athletic performance are vital in the dynamic environment of racket sports. For instance, perception and object tracking, structuring complicated visual information, voluntary attention shifts, and visuomotor coordination and speed are all components of visuospatial cognition (Possin, 2010; Spence & Feng, 2010). Racket athletes (table tennis, badminton) outperform nonathletes on fundamental visuospatial processing tests such as the attention network (Wang, Guo & Zhou, 2016), a cued RT task (Hung et al., 2004), a go/no-go visuospatial (Guo, Li & Yu, 2017), and attention and working memory (Wang et al., 2015). These results imply that long-term training in athletes under physical and cognitive loading may strengthen neural networks and plasticity, contributing to task-related information processing abilities (for review, see Nakata et al., 2010). The sport domain places strong demands on visuospatial processing in athletes (Scharfen & Memmert, 2019; Yarrow, Brown & Krakauer, 2009), and analysis of visuospatial processes in athletes can reveal the cognitive adaptation behind exceptional athletic performance. While previous research has compared experts and novices (Hung et al., 2004; Di Russo et al., 2006; Taddei et al., 2012; Wang, Guo & Zhou, 2016; Guo, Li & Yu, 2017), only a few studies have delved into the relationship between sports skill level (high/low) and cognitive processing performance. Furthermore, it is suggested that cognitive performance might be influenced by the level of skill expertise, such as distinguishing between high-performance and low-performance athletes. A meta-analysis by Scharfen & Memmert (2019) indicates that these differences present small to moderate effect sizes. Consequently, varying levels of expertise may influence cognitive performance outcomes. To elucidate this matter further, existing research has evidenced that a wider skill disparity between experts and novices correlates with the enhanced cognitive performance of the experts. However, the extent to which a narrowed skill gap between elite athletes and amateur competitors influences, or even maintains, the general visual cognitive processing advantage of elite athletes remains an area yet to be fully clarified. It is well-established that cognitive function benefits are prominent in the domain of sports, and these can contribute to optimizing sports training or identifying underlying talent potential.

Electrophysiological techniques, such as event-related potentials (ERP), provide the advantage of high temporal resolution for investigating the impact of athletic training on cognitive performance (Thompson et al., 2008). ERPs can assess implicit cognitive processing for stimulus coding and response execution (Luck, Woodman & Vogel, 2000). The P3 component of ERP has been utilized to study the hidden neuroelectric processing underlying cognitive performance in athletes and non-athletes. P3 amplitude is linked to updates in working memory concerning changes in mental representation (Donchin & Coles, 1988; Polich & Kok, 1995) and the allocation of attentional resources towards stimuli that are behaviorally relevant (Milne et al., 2013). On the other hand, P3 latency relates to the timing of stimulus classification (Kutas, McCarthy & Donchin, 1977). Studies have demonstrated that athletes tend to have a larger P3 amplitude compared to nonathletes in tasks such as action anticipation (Jin et al., 2011) and visuospatial memory (Chueh et al., 2017). These findings imply that prolonged athletic training may effectively channel attention resources towards action memory representation and attention processing, leading to quicker responses. Recent research has investigated the connection between different levels of sport skill (high skill-low skill) and cognitive activities (Huijgen et al., 2015; Lundgren et al., 2016; Verburgh et al., 2016). For instance, Vestberg et al. (2012) found that highly skilled soccer players outperformed less skilled soccer players in non-sports-related tasks (such as response inhibition, and cognitive flexibility). Similar studies have demonstrated that elite table tennis players have better cognitive performance than sub-elite table tennis players in inhibitory control tasks (Elferink-Gemser et al., 2018). Cognitive performance is modulated by sport skill levels (Lum, Enns & Pratt, 2002; Scharfen & Memmert, 2019), indicating that high skill athletes have better cognitive processing benefits and that basic cognitive functions can be improved through sports training. However, without measuring the underlying cortical mechanisms, these studies compare athletes with various skill levels. On the basis of these findings, we examined P3 amplitudes to determine whether elite athletic advantage in cognitive processing existed, in order to obtain insight into the potential brain mechanism behind the control process of elite athletes in visuospatial tasks.

The goal of this study was to evaluate the differences between skill level of sport (elite table tennis (ETT) players/amateur table tennis (ATT) players/nonathletes) on visuospatial attention and working memory task. We hypothesized that ETT may have superior visuospatial task performance (*i.e*., faster reaction times) than ATT, and that both athletes would perform better than nonathletes, as evidence suggests that through long-term sport training and extensive sport experience, a degree of cognitive function can be trained, which is beneficial for information processing performance (Voss et al., 2010). In addition, high-skill athletes have superior visuospatial-related cognitive performance compared to low-skill athletes (Scharfen & Memmert, 2019), and a greater component of P3 is associated with visuospatial cognition (Müller & Knight, 2002; Chueh et al., 2017), we hypothesized that if ETT is found to have superior visuospatial processing performance than ATT, and ETT may have a greater P3 amplitude than ATT, and that both athletes exhibit greater P3 amplitude than nonathletes when performing visuospatial cognitive tasks. In addition, to further understand whether there is a correlation between sport training and task performance or sport training and P3 components.

## Method

### Participants

Fifty-four participants were recruited by advertisement from universities in Taipei. This sample size was determined based on an *a priori* power analysis (alpha = 0.05, power = 0.80), and the effect size, *f* = 0.44 (Cohen, 1992), and similar to that seen in ERP studies investigating visuospatial attention and working memory in athletes (Chueh et al., 2017). They were divided into three groups based on their sport level: eighteen ETT players, eighteen ATT players, and eighteen nonathletes. ETT athletes (age 20.2 ± 2.4) were those who competed at the national division 1 level (top 3), and international competition experience (*n* = 10). ATT athletes (age 22.2 ± 2.2) had no experience competing at national division 1 level, but with national division 2 level experience (top 8). Nonathletes (age 20.7 ± 1.2) were those who had not engaged in regular physical activity as measured by the International Physical Activity Questionnaire (IPAQ) (Liou et al., 2008). Both levels of table tennis players maintained regular training for at least 6 months preceding their participation in the experiment. There were nine male and nine female participants in each group (Table 1). All the participants met the following criteria: (a) normal or corrected-to-normal visual acuity, (b) nonsmokers, (c) right-handed dominance, and (d) individuals did not report diagnosed psychiatric or neurological disorders, and take central nervous system medication. Prior to the start of the experiment, all participants were required to sign an informed consent approved from the Human Research Ethics Committee of National Taiwan Normal University (201602HM005).

**Table 1:** Demographics of the participants in each group (mean ± SD).

Variables	ETT (*n* = 18)	ATT (*n* = 18)	Nonathlete (*n* = 18)	Total (*n* = 54)
Female/Male	9/9	9/9	9/9	27/27
Right-handed	18	18	18	54
Age (years)	20.2 ± 2.4	22.2 ± 2.2	20.7 ± 1.2	21.0 ± 2.1
Height (cm)	168.6 ± 8.5	166.4 ± 7.1	168.4 ± 8.0	167.8 ± 7.8
Weight (kg)	61.6 ± 10.4	58.3 ± 10.8	58.7 ± 8.8	59.5 ± 10.0
TT training duration (hours)	3.3 ± 1.4	2.6 ± 0.6	–	3.0 ± 1.1
TT training frequency (session/week)	4.2 ± 3.8**	2.6 ± 2.6	–	3.4 ± 1.5
TT training experience (years)	12.0 ± 2.9**	8.1 ± 3.8	–	10.0 ± 3.9
Nonverbal IQ test	39.4 ± 4.0**	44.4 ± 4.8	45.8 ± 5.6	43.2 ± 5.4
Socioeconomic status of family	2.3 ± 0.9	2.6 ± 0.7	2.2 ± 0.6	2.4 ± 0.7
Total IPAQ (METs)	9,851.9 ± 6,416.8**	4,728.3 ± 3726.6	1,645.1 ± 1,182.2	5,408.4 ± 5,457.3
Video game experience (hours/week)	1.9 ± 3.5	2.1 ± 3.7	4.2 ± 5.9	2.7 ± 4.6

**Note:**

MET, Metabolic Equivalent. **Group effect at *p* < 0.01. Table tennis (TT) training reflects experience in the past 6 months.

### Cognitive assessments

**Figure 1:**
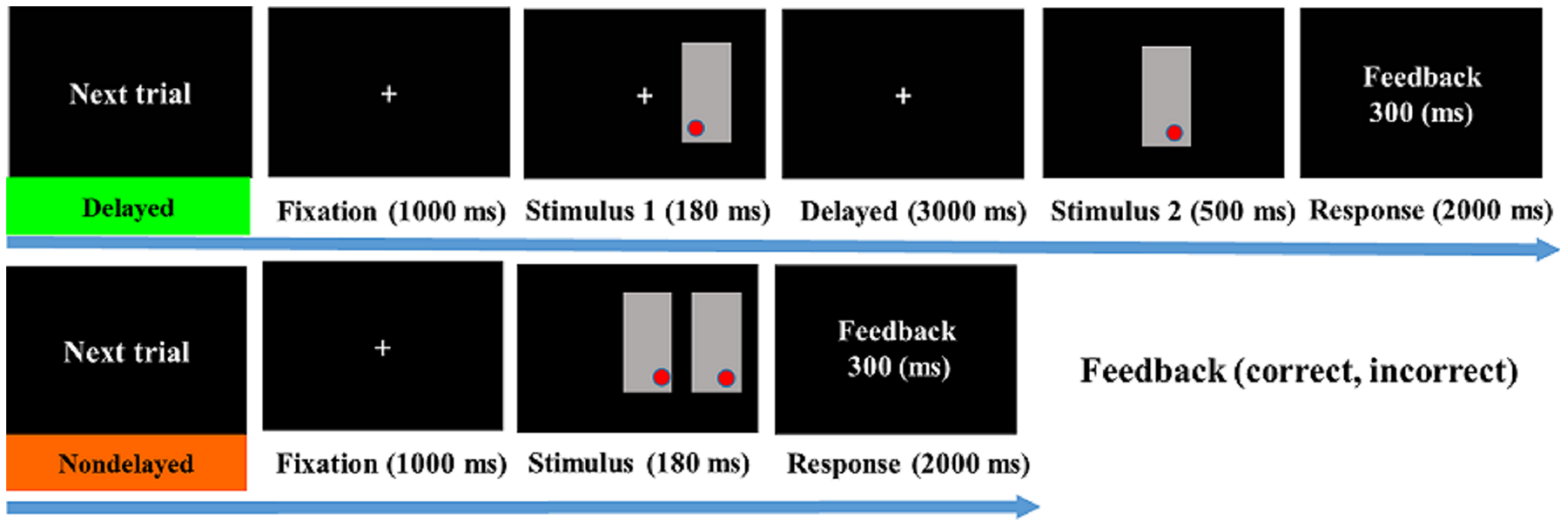
Illustration of the delayed match-to-sample and non-delayed task.

The current study employed a modified nondelayed and delayed match-to-sample tesk adapted from Müller & Knight (2002), Chueh et al. (2017) to assesses visuospatial attention (nondelayed condition) and visuospatial memory (delayed condition) (Fig. 1). The task was programmed with STIM 2.0 software (Neuroscan Ltd., El Paso, TX, USA). All stimuli were displayed on a 17-inch computer monitor with a black background positioned 60 cm in front of the subject. Similar to Chueh et al. (2017), the stimuli consisted of a red dot (0.5° × 0.5°) randomly presented within a 3.8° × 7.4° gray rectangle. A red dot could appear in any one of nine locations (*i.e*., center, center right, center left, upper center, upper right corner, upper left corner, lower center, lower right corner, and lower left corner) within its rectangle. Within the two rectangles, participants were required to determine whether the red dots were in the same or different spatial position. In the visuospatial attention condition (nondelayed condition), two rectangles were presented simultaneously, one in the centre and the other to the left or right of it. To avoid the potential effect of unwanted saccades, the two rectangle stimuli were presented for 180 ms, a duration typically shorter than voluntary saccade (Müller & Knight, 2002; Chueh et al., 2017). In the visuospatial memory condition (delayed condition), stimulus 1 (S1) was presented for 180 ms to the left or right of the central fixation (0.5° × 0.5°) with an equal probability of appearing on both sides, followed by a 3 s delay. Stimulus 2 (S2) was then presented for 500 ms in the centre of the screen. During the 3 s delay, participants were required to memorise the position of the S1 red dot and determine whether the position of the S2 red dot was the same or different. Before beginning the task, participants were reminded that both speed and accuracy were essential. When the red dot appeared in the same position, the left thumb pressed the “Yes” button, and when the red dot appeared in a different position, the right thumb pressed the “No” button. For the non-delayed and delayed conditions, the response time window was 2,000 ms, after which feedback (correct or incorrect) was provided based on the participant’s response. For the practise trials, each participant had to achieve a response accuracy of 80%. A total of 240 trials were divided into four blocks, with the non-delayed and delayed conditions being presented at random and with equal probability. Each block was separated by a 3–5 min rest period.

### Procedure

The experimenter concealed the aim of this study from the participants to preclude any psychological expectations that could introduce bias or distortion into the data. Stringent guidelines were enforced, such as prohibiting strenuous exercise, and consumption of food and drink (excluding water) for 1.5 h before the commencement of the experiment. Testing sessions were scheduled during daytime hours, and all experiments were concluded within an approximate 3-week period. Participants were required to complete the Socioeconomic Status of the Family (SES) inventory (Hollingshead & Redlich, 1958) and a non-verbal IQ test using Raven’s Progressive Matrices: SPM Plus Sets. This test, comprising a total of 60 items across five sets (with 12 items per set) (Styles, Raven & Raven, 1998), was overseen by researchers to evaluate abstract reasoning ability, ensuring that all participants possessed a normal IQ. They also completed the International Physical Activity Questionnaire (IPAQ) (Liou et al., 2008) and signed the informed consent form. Before the formal recording, participants were briefed on the cognitive task and were required to achieve an accuracy rate of 80 percent on practice trials. Upon completion of the experiments, participants were compensated with USD $30.

### Electroencephalographic recording

The electroencephalographic (EEG) recording procedure was similar to previous studies (Chueh et al., 2017). EEG activity was recorded at 30 electrode sites using an elastic electrode cap (Quick-Cap, Compumedics Neuroscan, Inc., 200 Charlotte, NC, USA). The electrode sites were mounted according to the modified International 10-10 System (Chatrian, Lettich & Nelson, 1985). Electrooculographic (EOG) activity is largely detected by monitoring both vertical and horizontal (HEOG and VEOG) activity, which is measured in the outer canthus and above and below the left orbit. The reference electrode was set to the mastoid average (A1, A2), and the ground electrode was set to FPz. All electrode impedances were below 5 kΩ. The EEG data acquisition was performed with a sampling rate of 1,000 Hz, using a DC-to 200-Hz filter and a 60-Hz notch filter.

### Event-related potential signal processing

The algorithm using (Semlitsch et al., 1986) was corrected the EOG activity and data reduction. The ERP analysis time epochs was defined from −100 ms of prestimulus to 1,000 ms of poststimulus onset, with the prestimulus 100 ms as baseline. The ERP data acquisition employed were bandpass filtered a bandpass filter (0.1–30 Hz cutoffs; 12 dB/oct). Epochs were exclusion of any electrode with an amplitude exceeding the ±50 μV criterion. Visual inspection of individual grand average ERP waveforms through inspection results and final peak detection analyses. In the nondelayed condition, the P3 amplitude was defined as the maximal positive peak (300 to 600 ms) after the stimulus onset, and as the maximal positive peak (300 to 600 ms) after the onset of the S2 stimulus in delayed condition. The P3 latency was defined according to the time point of the maximal P3 peak. P3 amplitude was distinguished across the midline electrodes (*i.e*., Fz, Cz, and Pz) (Johnson, 1993).

### Statistical analysis

One-way ANOVAs were separately computed to test homogeneity of the following demographic variables (DV): height, weight, non-verbal IQ, SES, levels of physical activity and video game experience among groups. An independent t-test was conducted to compare training experience and daily training hours, number of training sessions per week in the past 6 months and number of years in training between the two athlete groups.

Behavioral data analysis, 3 group (ETT players, ATT players, and nonathletes) × 2 condition (Nondelayed and Delayed) repeated measures ANOVAs were separately performed on behavioral data (*i.e*., response time, response accuracy, accuracy-adjusted response time) to examine group differences in behavioral performance. In addition, DV, such as IQ and physical activity, rather than a sport-specific training effect, were found to be nonhomogeneous (Green & Bavelier, 2003; Hackman & Farah, 2009). We hypothesize that the group effect might be influenced by IQ and physical activity. To investigate this, we conducted a Pearson correlation analysis to examine the relationship between task performance, DV (*e.g*., IQ, physical activity), accuracy-adjusted response time, and sport training. We measured overall task performance (accuracy and RT) using accuracy-adjusted response time (mean response time/accuracy) (Townsend & Ashby, 1983). This approach has been proposed to help rule out any possible effects of behavioral strategies on task performance (such as the speed-accuracy trade-off) (Getzmann, Falkenstein & Gajewski, 2013).

ERP data analysis, 3 group (ETT players, ATT players, and nonathletes) × 2 condition (Nondelayed and Delayed) × 3 electrode sites (Fz, Cz, and Pz) repeated measures ANOVAs were performed on the P3 amplitude and latency data separately. *Post hoc* comparisons were conducted using Tukey HSD significant difference tests. In this analysis, we conducted a covariate analysis of physical activity and IQ to test whether the physical activity and IQ is a confounding variables that may potentially bias the physical activity and IQ effect on P3 (Jaušovec & Jaušovec, 2000; McDowell et al., 2003). In addition, to test whether the P3 effect was due to a benefit of sport training (year, weekly frequency and hours), we conducted a Pearson correlation analysis to examine the relationship between the P3 amplitude/latency and sport training (ST). An alpha = 0.05 was set as the level of statistical significance for all analyses. Eta-squared effect sizes (η^2^) were reported for significant main effects and interactions, and a Greenhouse-Geisser correction was used to adjust for violations of the sphericity assumption. All data analyses was completed using IBM SPSS Statistics 23 (SPSS, Chicago, IL, USA).

## Results

### Demographic data

Table 1 presents the participants’ characteristics. No significant differences were observed in age (*F*(2, 51) = 2.863, *p* = 0.066), height (*F*(2, 51) = 0.420, *p* = 0.659), weight (*F*(2, 51) = 0.556, *p* = 0.577), handedness scores (*F*(2, 51) = 0.536, *p* = 0.589), video game experience (*F*(2, 51) = 1.387, *p* = 0.259), and socioeconomic status of the family (*F*(2, 51) = 1.579, *p* = 0.216) among groups. There was a significant difference in IQ (*F*(2, 51) = 9.061, *p* < 0.01, η^2^ = 0.262), and a *post hoc* comparison revealed that ETT players had a lower IQ than ATT players (*p* < 0.003) and nonathletes (*p* < 0.001), but no significant difference between the ATT and nonathletes (*p* < 0.368) was observed.

With regards to sport characteristics, there was a significant difference between the two athlete groups in terms of the number of years engaged in sport training (*t*(34) = 3.411, *p* < 0.01, d = 1.14) and weekly training frequency within the past 6 months (*t*(34) = 3.972, *p* < 0.01, d = 1.32), the results show that ETT players had longer training frequency and training experience (years) than ATT players. There was no difference in the number of daily training hours within the previous 6 months (*t*(34) = 1.966, *p* = 0.057). ETT players had greater physical activity levels than ATT players, and both athlete groups had greater levels of physical activity than nonathletes (*F*(2, 51) = 16.436, *p* < 0.01, η^2^ = 0.392).

### Behavioral data

#### Accuracy data

Table 2 presents the results for mean correct response accuracy. The data showed a main effect for condition (*F*(1,51) = 89.66, *p* < 0.01, η^2^ = 0.637), with a higher accuracy in the nondelayed condition (96.10%) than in the delayed condition (90.17%). Neither an effect of condition by group (*F*(2,51) = 0.943, *p* = 0.396, η^2^ = 0.036) nor a main effect for group (*F*(2, 51) = 1.250, *p* = 0.295, η^2^ = 0.047) was observed.

**Table 2:** Behavioral performance results for each group.

Condition	ETT	ATT	Nonathlete
3 s-delayed RA nondelayed RA	89.08 ± 4.17	91.08 ± 4.21	90.33 ± 4.20
	96.12 ± 3.03	96.89 ± 2.26	95.27 ± 3.61
3 s-delayed RT nondelayed RT	617.98 ± 97.58*	638.84 ± 64.95	713.83 ± 129.13
	674.30 ± 74.41*	682.80 ± 72.42	762.80 ± 111.41

**Note:**

Response accuracy (%); RT, Response time (ms); mean ± SD *Group effect at *p* < 0.05.

#### Reaction time data

Table 2 shows the mean correct reaction time and accuracy-adjusted RT (AART) across groups and conditions. There was no significant main effect of group × condition interaction effect (*F*(2,51) = 0.193, *p* = 0.825, η^2^ = 0.008). However, a significant main effect of condition, (*F*(1,51) = 37.141, *p* < 0.01, η^2^ = 0.421), revealed that reaction times in the delayed condition (656.89 ms) was shorter than in the nondelayed condition (706.63 ms), and main effect of group (*F*(2, 51) = 5.494, *p* < 0.05, η^2^ = 0.177) and both ETT players (646.15 ms, *p* < 0.003) and ATT players (660.83 ms, *p* < 0.012) demonstrated shorter reaction times than nonathletes (738.32 ms) regardless of task conditions, but there was no difference between the two athlete groups (*p* < 0.625) was observed. Similar results were found for RT and accuracy-adjusted RT, with a significant effect of group (*F*(2,51) = 5.238, *p* < 0.01, η^2^ = 0.170). Both athlete groups had a shorter accuracy-adjusted RT than the nonathletes regardless of condition, but there was no difference between the two athlete groups (ETT: 7.00 ms/% & ATT: 7.04 ms/% < nonathletes: 7.94 ms/%). There were no significant effects of condition by group (*F*(2,51) = 0.038, *p* = 0.963, η^2^ = 0.001), and condition (*F*(1,51) = 0.271, *p* = 0.605, η^2^ = 0.005).

### P3 data

**Figure 2:**
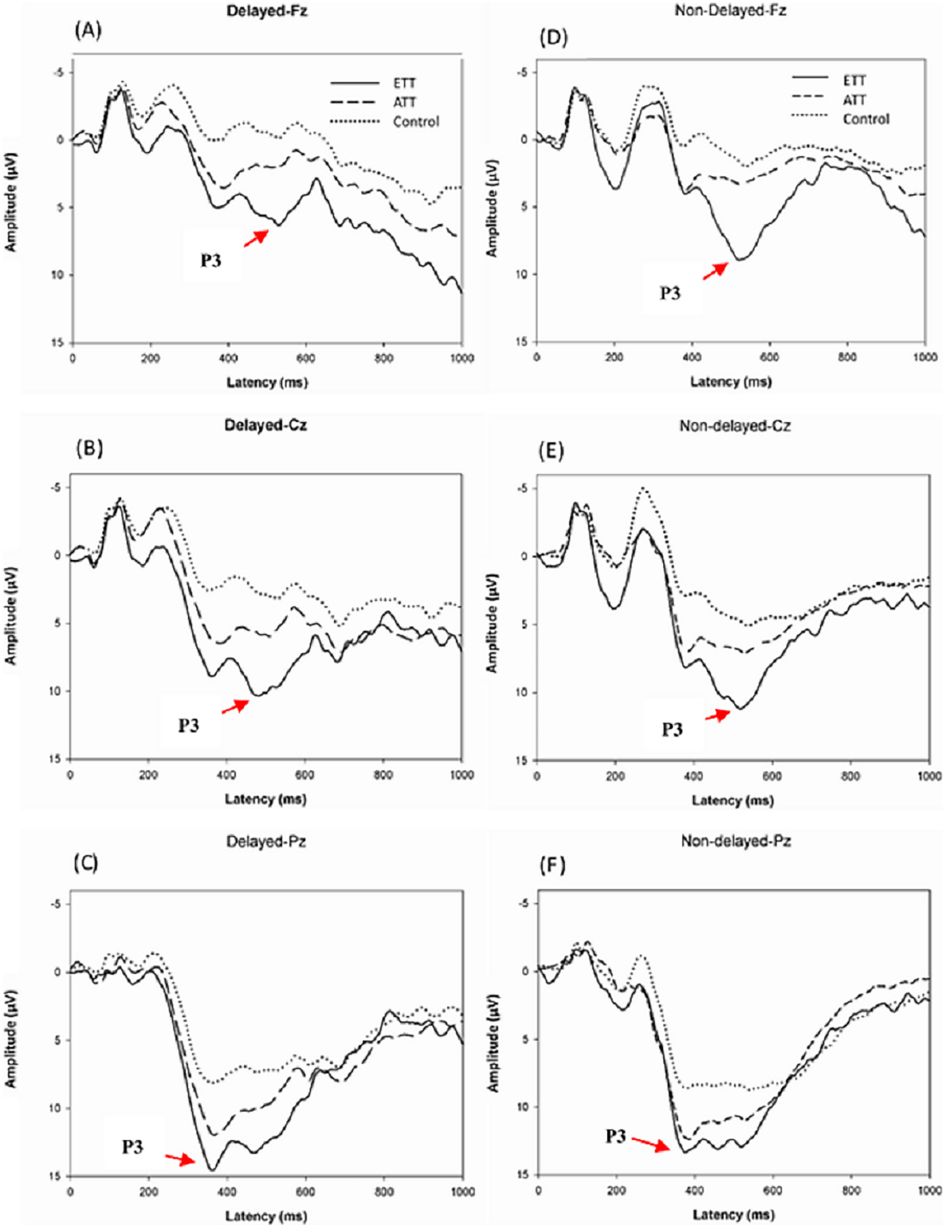
Grand average ERP at the Fz, Cz, and Pz sites stratified by group for the Delayed condition (A–C) and the Nondelayed (D–F) condition.

Figure 2 illustrates the grand average ERP results at Fz, Cz, and Pz for each group and each condition. Table 3 shows the means and standard deviations P3 across groups and conditions. In the analysis of P3 response latency to target stimuli, the analysis did not show an interaction effect for group × condition × electrode site (*F*(4,51) = 1.811, *p* = 0.133, η^2^ = 0.069), group × condition (*F*(2,51) = 1.207, *p* = 0.308, η^2^ = 0.047), group × electrode site (*F*(4,51) = 0.898, *p* = 0.440, η^2^ = 0.035), or condition × electrode site (*F*(2,51) = 0.485, *p* = 0.616, η^2^ = 0.010) or a main effect of group (*F*(2,51) = 0.616, *p* = 0.544, η^2^ = 0.025), or main effect of condition (*F*(1,51) = 0.367, *p* = 0.548, η^2^ = 0.007), or electrode site (*F*(2,51) = 0.742, *p* = 0.433, η^2^ = 0.015).

**Table 3:** P3 at the Fz, Cz, and Pz sites stratified by group for the delayed condition and the nondelayed condition in each group (mean ± SD).

		ETT		ATT		Nonathlete	
	Site	Delayed	Nondelayed	Delayed	Nondelayed	Delayed	Nondelayed
P3L	Fz	422.68 ± 77.30	463.33 ± 74.40	443.06 ± 90.40	441.00 ± 74.40	407.167 ± 83.33	441.88 ± 96.04
P3L	Cz	432.94 ± 70.12	473.50 ± 69.12	445.72 ± 74.19	466.68 ± 82.08	427.94 ± 92.90	455.00 ± 91.96
P3L	Pz	389.00 ± 45.70	426.68 ± 79.06	417.44 ± 62.77	420.389 ± 61.99	420.44 ± 84.65	435.44 ± 89.95
P3A	Fz	8.10 ± 6.73	10.69 ± 11.08	6.05 ± 3.23	5.88 ± 3.54	1.60 ± 3.74	2.52 ± 4.16
P3A	Cz	12.21 ± 4.12	13.26 ± 5.20	8.72 ± 3.81	9.99 ± 3.66	5.12 ± 4.49	6.20 ± 4.62
P3A	Pz	15.48 ± 4.57	15.98 ± 4.42	12.16 ± 3.36	14.05 ± 4.45	9.70 ± 3.36	11.27 ± 4.47

**Note:**

L, Latency (ms); A, Amplitude (µV). site, electrode. The main effect of group (*F*(2, 51) = 4.756, *p* < 0.01, η^2^ = 0.229) revealed that the ETT players (12.61 µV) showed greater P3 amplitudes than the ATT players (9. 64 µV, *p* < 0.030) and nonathletes (6.07 µV, *p* < 0.001), and ATT players (*p* < 0.007) and had a greater P3 amplitude than nonathletes.

In the analysis of P3 response amplitude to target stimuli, there was no significant interaction effect of group × condition × electrode (*F*(4,51) = 0.569, *p* = 0.621, η^2^ = 0.034), condition × group (*F*(2,51) = 0.215, *p* = 0.808, η^2^ = 0.013), group × electrode (*F*(2,51) = 0.303, *p* = 0.762, η^2^ = 0.019), condition × electrode (*F*(2,51) = 0.413, *p* = 0.584, η^2^ = 0.013), electrode (*F*(1,51) = 0.991, *p* = 0.335, η^2^ = 0.030), or condition (*F*(1,51) = 0.140, *p* = 0.711, η^2^ = 0.004). Finally, a significant main effect of group (*F*(2, 51) = 4.756, *p* < 0.01, η^2^ = 0.229) was observed, the results revealed that ETT players (12.61 **µV**) had a greater P3 amplitude than ATT players (9. 64 **µV**, *p* < 0.030) and nonathletes (6.07 **µV**, *p* < 0.001), and ATT players (*p* < 0.007) and had a greater P3 amplitude than nonathletes.

### Correlation between accuracy-adjusted RT and IQ

AART and IQ analysis, we found that the AART did not correlate with IQ in either the attention (*r* = 0.134, *p* = 0.334) or in the working memory condition (*r* = 0.264, *p* = 0.054) across subjects, indicating that IQ may not directly account for the group effect of AART in the present study.

### Correlation between AART and physical activity levels

AART and physical activity levels (PAL) analysis, we found that the AART did not correlate with PAL in either the attention (*r* = −0.267, *p* = 0.051) or in the working memory condition (*r* = −0.262, *p* = 0.056) across subjects, indicating that levels of physical activity may not directly account for the group effect of task performance in the present study.

### Correlation between AART and ST

In addition, AART and ST analysis, We found that the AART did not correlate with years engaged in table tennis training in either the attention (*r* = 0.269, *p* = 0.112) or in the working memory condition (*r* = 0.177, *p* = 0.302), with weekly training frequency in either the attention (*r* = −0.079, *p* = 0.651) or in the working memory condition (*r* = 0.064, *p* = 0.710) and with training number of hours in either the attention (*r* = −0.114, *p* = 0.509) or in the working memory condition (*r* = 0.082, *p* = 0.636) across subjects. This suggests that the sport training experience (years), weekly training frequency and hours may not directly account for the group effect of AART in this study.

### ERP correlation analysis

**Figure 3:**
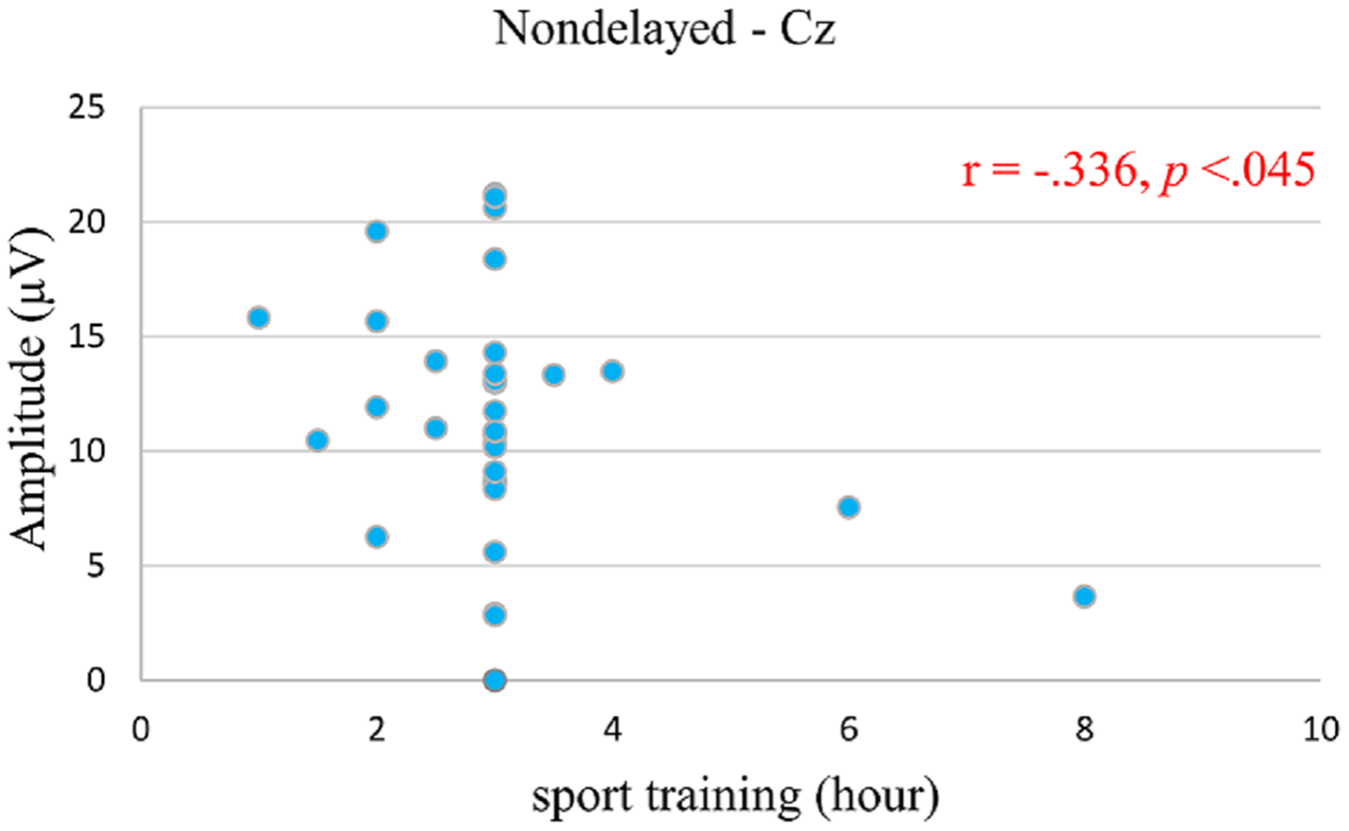
Correlations sport training (hour) behavioral and P3 amplitude.

For the analysis of P3 amplitude and ST years, we observed no correlation with ST years in the attention condition at Fz (*r* = 0.067, *p* = 0.698), Cz (*r* = 0.133, *p* = 0.439), and Pz (*r* = 0.201, *p* = 0.240), or in the working memory conditions at Fz (*r* = 0.140, *p* = 0.415), Cz (*r* = 0.252, *p* =0.138), and Pz (*r* = 0.201, *p* = 0.240). Similarly, when analyzing P3 amplitude and weekly practice frequency, no correlation was found in the attention condition at Fz (*r* = 0.057, *p* = 0.743), Cz (*r* = 0.018, *p* = 0.918), and Pz (*r* = 0.047, *p* = 0.787), or in the working memory conditions at Fz (*r* = −0.021, *p* = 0.904), Cz (*r* = 0.060, *p* = 0.728), and Pz (*r* = 0.120, *p* = 0.486). These findings suggest that neither weekly practice frequency nor ST years directly influence the group effect of P3 amplitude in this study. For the P3 amplitude and ST hours analysis, no correlation was observed in the attention condition at Fz (*r* = −0.078, *p* = 0.652) and Pz (*r* = −0.268, *p* = 0.114), or in the working memory conditions at Fz (*r* = −0.128, *p* = 0.457), Cz (*r* = −0.168, *p* = 0.326), and Pz (*r* = −0.117, *p* = 0.497). However, a negative correlation was noted for P3 amplitude at Cz (*r* = −0.336, *p* < 0.045) (Fig. 3) in the attentional conditions, indicating that athletes with higher training hours utilize fewer neural resources, while those with lower training hours use more.

Regarding the P3 latencies and ST years analysis, no correlation with ST years was found in the attention condition at Fz (*r* = 0.123, *p* = 0.474), Cz (*r* = 0.197, *p* = 0.251), and Pz (*r* = −0.003, *p* = 0.987), or in the working memory conditions at Fz (r = 0.150, *p* = 0.382), Cz (r = 0.148, *p* = 0.390), and Pz (*r* = −0.080, *p* = 0.641). Similarly, for the P3 latencies and weekly practice frequency analysis, no correlation was observed in the attention condition at Fz (*r* = 0.036, *p* = 0.835), Cz (*r* = 0.067, *p* = 0.699), and Pz (*r* = −0.191, *p* = 0.264), or in the working memory conditions at Fz (*r* = −0.083, *p* = 0.631), Cz (*r* = −0.150, *p* = 0.382), and Pz (*r* = −0.134, *p* = 0.434). For the P3 latencies and ST hours analysis, no correlation was found in the attention condition at Fz (*r* = 0.121, *p* = 0.481), Cz (*r* = 0.082, *p* = 0.636), and Pz (*r* = −0.322, *p* = 0.056), or in the working memory conditions at Fz (*r* = 0.096, *p* = 0.577), Cz (*r* = −0.056, *p* = 0.745), and Pz (*r* = 0.021, *p* = 0.904). These results suggest that ST hours, weekly practice frequency, and ST years may not directly influence the group effect of P3 latencies in this study.

## Discussion

This study aimed to examine the moderating effect of sport skill levels (ETT, ATT, and nonathletes) on behavioral and neuroelectric performance in fundamental visuospatial tasks. To address this issue, we modified non-sports-related cognitive tasks (Müller & Knight, 2002) to assess the disparities between visuospatial attention and working memory of athletes and nonathletes with varying levels of sport skill. In addition, the neural mechanism of athletes with varying levels of competence was investigated. Table tennis players exhibited reduced reaction times and accuracy-adjusted RT than nonathletes in both the visuospatial attention and memory conditions, regardless of their skill level. However, there was no difference between the two athlete groups. In addition, independent of task conditions, ETT demonstrated a greater P3 amplitude than ATT, and both athletes demonstrated a greater P3 amplitude than non-athletes. The results demonstrate that table tennis players exhibit significantly enhanced visuospatial abilities under all tested conditions. Furthermore, the ETT data suggest this superiority may be attributed to the efficient allocation of neural resources during the processing of visuospatial information. Additionally, we found a negative correlation between P3 amplitude and daily training hours in the attention condition. This suggests that a longer daily training duration might result in a smaller P3 amplitude, while a shorter training duration might lead to a larger P3 amplitude.

Regardless of task condition, the RT of both levels of table tennis players was shorter than that of nonathletes. These findings are consistent with those of previous research utilizing non-sport-related visual-spatial tasks (Wang et al., 2015; Wang, Guo & Zhou, 2016; Chueh et al., 2017; Guo, Li & Yu, 2017). These results suggest that table tennis players (average ETT 654.67 ms and average ATT 655.6 ms) are quicker than nonathletes at identifying information matching and selecting responses (average 734.06 ms). Compared to nonathletes, table tennis players have better attention deployment when performing tasks of uncertain stimulus and matching identification, which facilitates faster response selection (Hung et al., 2004; Guo, Li & Yu, 2017). However, visual spatial processing may be improved through sports training (Voss et al., 2010). Current findings indicate that table tennis players respond more quickly than nonathletes to general visuospatial tasks, supporting the broad transfer hypothesis and the notion that training specific cognitive skills can enhance performance on cognitively relevant untrained cognitive tasks (Furley & Memmert, 2010, 2011; Voss et al., 2010).

By evaluating P3 latency, the current study found no statistically significant differences between groups. Comparing athletes and nonathletes (Chueh et al., 2017) and physical activity participants and sedentary controls on comparable visuospatial tasks, this result was consistent with previous research (Wang & Tsai, 2016). Interestingly, previous research has found no significant difference in the P3 latency between athletes and non-athletes; however, a group difference was observed in the P3 amplitude during sport-specific and non-sports-related visuospatial attentional cueing tasks (Chueh et al., 2017; Wang & Tu, 2017). Thus, we hypothesize that table tennis players improve visuospatial cognitive performance by modulating the allocation of neural resources to task-relevant stimuli, but not by enhancing neural processing speed during stimulus evaluation/classification.

However, the present study went one step further by demonstrating that the enhanced P3 amplitude was linearly related to the level of table tennis expertise. Specifically, we observed that ETT players showed the largest followed by ATT, with nonathletes showing the smallest amplitude. Greater P3 amplitudes reflect the amount of attention (Pontifex, Hillman & Polich, 2009) and allocation of attention resources to behavior related stimuli (Milne et al., 2013). This finding is consistent with a previous study showing that athletes (open skill and closed skill) exhibit greater P3 amplitudes in visuospatial memory tasks compared to nonathletes (Chueh et al., 2017), this suggests that sports training may be promote to cognitive domains with higher mental loads (such as memory) by modulating the allocation of neural resources to task-related stimuli. Similar results were found for racket sports, indicating that badminton players responded faster and had a greater P3 amplitude than nonplayers in the action anticipation task (Jin et al., 2011; Wang & Tu, 2017). The authors interpreted the P3 amplitude to indicate that players may devote more attention resources to memory representations associated with badminton, which is conducive to action anticipation processing speed. In addition, Peng et al. (2022) demonstrated that table tennis players had superior spatial cognitive processing and task performance than nonathletes, and that the P3 amplitude was substantially greater in the right hemisphere than the left. This suggests that the advantage of table tennis players is due to the neural activity of the right hemisphere, which is conducive to spatial location processing with attention. Moreover, these findings are consistent with current research, in which it was demonstrated that ETT athletes elicited a greater P3 amplitude than ATT athletes, suggesting that they devote more neural resources to the visuospatial attention and memory task. This indicates that table tennis players have a more effective deployment of their attention, which reflects a greater allocation of neural resources (Hung et al., 2004). The present study corroborates and extends upon the findings of Scharfen & Memmert (2019), demonstrating that the level of sports skill significantly moderates cognitive function, with observed effect sizes ranging from small to medium. A notable group effect was detected (η^2^ = 0.229), highlighting a pronounced impact on P3 amplitude among table tennis players (ETT, 12.61 µV > ATT, 9.64 µV > nonathletes, 6.07 µV). This underscores the capacity of athletes in sports groups to effectively modulate neural resource allocation. The enhanced structural and functional brain plasticity associated with prolonged training in specialized sports, such as table tennis, potentially augments foundational visuospatial processing capabilities (Zhang et al., 2021). The distinctive traits of table tennis players—rapid response times, precise motor coordination, and specialized cognitive processing (Gu et al., 2021; Guo, Li & Yu, 2017)—are pivotal to their superior performance, underscoring the integral relationship between fundamental visuospatial skills and increased neural resource processing. This relationship is further affirmed by the modulation of neural resources contingent upon sports skill level.

Moreover, this study employed correlation analysis to explore the nexus between sports training and P3 components more deeply. A negative correlation was observed between Cz-P3 amplitude and daily training hours under attention conditions, indicating that extended training durations may correspond to diminished attentional resources, as evidenced by reduced Cz-P3 amplitude. Conversely, shorter training durations were associated with larger Cz-P3 amplitudes, suggesting an increase in attentional resources. This finding diverges from previous observations and posits that athletes might experience cognitive adaptation through prolonged sports training, which is integral for optimizing sport performance (Furley & Memmert, 2011; Furley, Memmert & Schmid, 2013). However, the potential for a cognitive plateau—wherein the benefits of training plateau or diminish beyond a certain threshold (Lampit et al., 2014)—emerges with excessive training. Symons, Bruce & Main (2023) and Xu et al. (2022) have suggested that adjustments in training frequency are necessary to sustain training effects, especially in the face of risks such as cognitive plateau (decline) and the impacts of sleep deprivation, including decreased P3 amplitude. The empirical evidence suggests that table tennis players, averaging 3–5 h of training daily, may encounter significant physical and cognitive fatigue due to the demanding nature of the sport. Thus, the potential detriments of prolonged high-intensity training might counterbalance the cognitive benefits of such activities. To circumvent these challenges, athletes are advised to judiciously manage their training regimens by modulating intensity, duration, and frequency to preserve and enhance cognitive processing capabilities, thereby ensuring peak athletic performance (Symons, Bruce & Main, 2023). Nonetheless, the interpretations drawn from our correlational analysis should be approached with caution. While these associations are compelling, they do not establish causality. Future investigations are warranted to verify these relationships and elucidate the mechanisms underlying the interaction between training variables (frequency, intensity, duration) and P3 amplitude among table tennis athletes.

Some research limitations and future recommendations should be mentioned. Firstly, while our research demonstrates that table tennis players possess superior fundamental visuospatial processing capabilities, it raises the question of whether individuals with naturally stronger visuospatial cognitive functions are more inclined to excel in sports training. Due to the inherent limitations of the cross-sectional approach adopted in this study, the causal relationship between sports training and enhanced cognitive performance remains ambiguous. Consequently, there is a compelling need for future investigations to employ longitudinal methodologies. Such studies should aim to establish a definitive causal link by tracking cognitive changes in individuals who undergo specialized table tennis training, compared to those without such training, in a controlled and randomized setting. Second, previous research has demonstrated that table tennis players have superior cognitive functions, including response inhibition (You et al., 2018), attentional flexibility (Hung et al., 2004; Wang, Guo & Zhou, 2016), and visuo-perceptual abilities (for a meta-analysis, see Scharfen & Memmert, 2019). This study was limited to the relationship between an athlete’s talent level and visual-spatial tasks and was unable to summarize whether there are differences in other cognitive function performance and neural activity mechanisms, which requires further investigation. Third, previous research has demonstrated that gender influences visuospatial processing (Hugdahl, Thomsen & Ersland, 2006). A recent meta-analysis suggested that participants measuring cognitive function were predominantly male athletes, and the absence of female athletes may have reduced the specificity of detection (Scharfen & Memmert, 2019), with some studies recruiting only a single gender (Wang et al., 2015; Wang & Tu, 2017). It can be observed that the gender of recruited athletes is inconsistent, which may be one of the factors contributing to the variable cognitive performance. The current study recruited an equal number of male and female participants for each group, but it must be determined in the future whether gender influences cognitive performance. Finally, while this study focused on athletes’ training years, weekly practice frequency and daily hours over the past 6 months, this timeframe might be limited. Future research should delve deeper into long-term training methodologies, encompassing aspects like intensity, duration, frequency, training plans, and the nature of training specifically, whether it includes cognitive tasks (Nakata et al., 2010). Such studies could further explore how these training methods influence sport skill levels.

## Conclusions

In conclusion, our research findings highlight that table tennis players, irrespective of their proficiency level, exhibit enhanced processing speed in visuospatial tasks compared to nonathletes. The study also sheds light on the neural underpinnings in table tennis players, with a particular emphasis on the augmented neural resource allocation. Regardless of task conditions, the observation of enhanced P3 amplitudes in athletes with higher skill levels, as compared to their lower-skilled counterparts, implies a more substantial allocation of neural resources during visuospatial tasks. Our findings provide substantial support for the broad transfer hypothesis, indicating that the skills acquired by table tennis players in their specialized sport may not only be confined to the sport itself but also potentially have a positive impact on visuospatial attention and memory performances.

## Supplemental Information

10.7717/peerj.17295/supp-1 Supplemental Information 1 Raw data includes demographics, behaviors, and ERP data for each group. group 1 = ETT, 2 = ATT, 3 = Nonathlete

## Additional Information and Declarations

### Data Availability

The following information was supplied regarding data availability: The raw data is available in the Supplemental File.
